# Comparison of Endoscopic Ultrasound and Transabdominal Ultrasound in the Detection of Gallbladder and Common Bile Duct Microlithiasis

**DOI:** 10.7759/cureus.58756

**Published:** 2024-04-22

**Authors:** Khurshid Ul Hassan Khurshid, Rashk e Hinna, Rao Saad Ali Khan, Arshman Rauf Asghar, Aalia Mushtaq Chaudhary, Muhammad Afzal, Uzair Ali Khan, Zoya Ali Khan, Ayaan Ali Khan, Rao Zaid Ali Khan

**Affiliations:** 1 Gastroenterology, Pak Emirates Military Hospital, Rawalpindi, PAK; 2 Internal Medicine, Combined Military Hospital (CMH) Lahore Medical College and Institute of Dentistry, Lahore, PAK; 3 Medical Oncology, Fauji Foundation Hospital, Rawalpindi, PAK; 4 Internal Medicine, Margalla Institute of Health Sciences, Rawalpindi, PAK; 5 Health Sciences, The Downtown School, Seattle, USA; 6 Science, Lahore Grammar School, Lahore, PAK; 7 Health Sciences, International Community School, Kirkland, USA; 8 Basic Sciences, Brown University, Providence, USA

**Keywords:** gallbladder stones, endoscopic retrograde cholangiopancreatography (ercp), cholecystectomy, transabdominal ultrasound, endoscopic ultrasound (eus), microlithiasis

## Abstract

Objective: Endoscopic ultrasonography (EUS) is an emerging method with a wide range of potential uses in gastroenterology, including the detection of bile duct stones and the identification of early ductal alterations in suspected patients. This study was designed to compare the diagnostic yield of EUS and transabdominal ultrasound (TUS) in the detection of gallbladder and common bile duct (CBD) microlithiasis.

Method: Patients with biliary colic with normal initial TUS were the subjects of this prospective study. EUS scan was performed on all recruited patients and linear endoscopes were used for the EUS examination. Cholecystectomy and histological analysis were done in patients within two weeks after EUS revealing cholelithiasis whereas the cases of CBD stone/microlithiasis were confirmed by endoscopic retrograde cholangiopancreatography (ERCP). The mean values of all hematological characteristics were independently determined for males and females and then compared using Student's t-test. For statistical significance, a p-value of 0.05 or below was used.

Results: A total of 131 patients, including 77 females and 54 males, with a mean age of 38.41 ± 14.78 years were examined. All 78 (59.5%) individuals who had cholecystectomy were found to have gallstones or microlithiasis as successfully diagnosed by EUS. The sensitivity and specificity of EUS were 92.9% and 100%, respectively, for CBD stones and 98.8% and 100%, respectively, for the detection of gallbladder microlithiasis. The agreement between EUS and TUS was fair for CBD stones (κ = 0.214) and very weak for microlithiasis (κ = -0.093).

Conclusion: EUS demonstrates a superior yield over TUS in detecting gallbladder stones and CBD microlithiasis, offering a more reliable diagnostic modality.

Limitation: This was a single-center study.

## Introduction

The technological advancements of the 20th century have positioned ultrasound (US) as a pivotal discovery that has transformed the field of diagnostic imaging. This diagnostic imaging has helped in the detection of various prevalent gallbladder diseases, with cholelithiasis being the most frequently encountered condition, affecting approximately 10-15% of adults [1]. Around 5% of other conditions, such as gallbladder polyps, are found throughout the world [1]. Gallbladder cancer has a relatively low global incidence, approximately 2/100,000 individuals [2]. Ultrasound is particularly useful in the imaging of the gallbladder due to its ability to provide real-time, non-invasive images of the organ and surrounding structures. In addition to ultrasound, other imaging modalities such as computed tomography (CT), magnetic resonance imaging (MRI), endoscopic ultrasonography (EUS), and endoscopic retrograde cholangiopancreatography (ERCP) are also utilized in the diagnosis and management of gallbladder diseases. These methods provide additional information that complements each other and are frequently used together to thoroughly assess the gallbladder and aid in making treatment decisions [3].

For the evaluation of gallstones, transabdominal ultrasound (TUS) is the preferred technique, offering a diagnostic accuracy of 95%. However, its effectiveness diminishes in cases involving obese patients, meteorism, gallstones smaller than 5 mm, and cystic stones [4]. Similarly, using TUS to visualize the common bile duct (CBD) for diagnosing sludge or microlithiasis is highly insensitive, at approximately 55%, and lacks specificity [5]. When there is a high clinical suspicion of gallstones despite a negative TUS, it is advisable to perform an EUS [4]. It is gaining more popularity as a diagnostic tool [6] and therapeutic modality in the treatment of pancreaticobiliary diseases, and more specifically, pancreaticobiliary malignancies due to the emergence of the new field of interventional EUS [7,8]. It offers high-resolution imaging for assessing gastrointestinal wall diseases and nearby organs like the pancreas, bile duct, and liver. Widely used in adults since the early 1980s, it aids in diagnosing, treating, and monitoring diseases affecting these organ systems [6]. EUS offers greater sensitivity than abdominal ultrasound because it is closer to the gallbladder, operates at a higher frequency, and is totally safe. Previous studies exploring EUS efficacy in patients with biliary colic and normal abdominal ultrasounds indicate potential benefits in lesion detection. However, these studies are constrained by small sample sizes, incomplete clinical symptom descriptions, and evaluations not limited to microlithiasis and biliary sludge, but also including cholelithiasis and choledocholithiasis [4].

Numerous studies have provided evidence that EUS may assist in the diagnosis of numerous gallbladder and related diseases. Therefore, this study was designed to evaluate the indicative precision of EUS for gallstones and CBD microlithiasis in individuals with a normal TUS.

## Materials and methods

Study design

It was a cross-sectional observational study. Data were collected prospectively. A total of 210 patients were reported to the outpatient Gastroenterology Department of the Pak Emirates Military Hospital (PEMH), Rawalpindi, Pakistan from emergency and through referrals from various hospitals for the treatment of biliary-type stomach discomfort and/or acute pancreatitis (of unknown cause) between March 2022 and February 2023.

Inclusion criteria

Patients who were aged nine years and above, fit and willing to undergo both EUS and TUS examinations, gave informed consent for participation, and with no evidence of gallstones or microlithiasis on the initial screening test (TUS) were selected for the study.

Exclusion criteria

Patients having acute pancreatitis caused by factors other than gallbladder or CBD stone, stricture in their pancreatic duct, and who already had cholecystectomy were excluded from the study. Also, patients who had recovery time from pancreatitis shorter than four weeks were not considered for our study.

Sample collection

A comprehensive set of tests, including complete blood cell count, liver function test, international normalized ratio, and serum amylase, was conducted in the initial phase of diagnosis. Following this, each patient underwent examination by a specialized radiologist using TUS. If no stones were found or if the biliary scans were inadequate during the initial scan, which took place during the acute phase of the illness, ultrasonography was repeated once. After the acute pancreatitis episode had resolved for at least four weeks and the biliary-type stomach discomfort had diminished, EUS was performed on these patients.

Procedure

All 131 participants were scheduled to undergo linear EUS under intravenous midazolam sedation, with the patient positioned in the left lateral decubitus position. Throughout the procedure, patients were closely monitored, and their oxygen levels were measured using a pulse oximeter. The gallbladder and bile duct were imaged using ultrasound by capturing images of the duodenum's first and second segments, along with the distal antrum and pylorus. Multiple images of the biliary system were captured by adjusting the probe's position. Stones, microlithiasis, and other diseases were identified. Cholecystectomy was recommended for patients with gallbladder wall thickness diagnosed by EUS (>3 mm), as well as biliary microlithiasis or stones 3 mm or larger. After cholecystectomy, the removed tissue was sent for histological analysis to confirm the presence of microlithiasis and/or stones. Patients with dilated CBD or biliary sludge were also successfully confirmed by ERCP. All cholecystectomies were performed using laparoscopic techniques, and patients were continuously monitored post procedure for any signs of recurrence of sludge/calculi in the CBD.

Ethical approval

This study was approved by the Research Ethical Committee of the institution, and all patients gave their informed, written consent before participating, by the ethical principles of the 1975 Declaration of Helsinki.

Data analysis

The categorical data were presented as frequency and percentages whereas continuous data were presented as mean and standard deviation. We calculated the mean values of all hematological attributes for males and females separately and compared their mean values using Student's t-test. P-values less than 0.05 were considered statistically significant. Receiver operating characteristic (ROC) curve, sensitivity, specificity, positive predictive value (PPV), and negative predictive value (NPV) were drawn for both EUS and TUS with cholecystectomy and histology for gallstones and ERCP for CBD stones as a gold standard for reference. Likewise, kappa coefficients were computed to assess concordance between EUS and TUS. Kappa values of 0 or less mean there is no agreement. Values between 0.01 and 0.20 suggest slight agreement. The fair agreement is indicated by values from 0.21 to 0.40, while moderate agreement falls between 0.41 and 0.60. Substantial agreement is seen with values from 0.61 to 0.80, and almost perfect agreement is represented by values from 0.81 to 1.00.

## Results

Out of the 131 patients, 54 (41.2%) were males and 77 (58.8%) were females, with a mean age of 38.41 ± 14.8 years. A total of 107 (81.7%) patients had reported one or more episodes of biliary-type abdominal pain and 38 (29%) had one or more episodes of acute pancreatitis. The EUS findings are summarized in Table 1.

**Table 1:** Clinical and radiological attributes of the participants CBD: common bile duct; ERCP: endoscopic retrograde cholangiopancreatography.

Variables		Frequency (n)	Percentage (%)
Gender	Male	56	42.7
	Female	75	57.3
Biliary abdominal pain	Yes	107	81.7
	No	24	18.3
Acute pancreatitis	Yes	38	29
	No	93	71
Gall bladder stones (<3mm)	Yes	1	0.8
	No	130	99.2
Gall bladder stones (≥3mm)	Yes	78	59.5
	No	53	40.5
CBD microlithiasis (<3mm)	Yes	2	1.5
	No	129	98.5
CBD stones (≥3mm)	Yes	27	20.6
	No	104	79.4

There were different parameters tested in all patients recruited for this study other than age and body mass index (BMI). After assessment, the average levels of alanine transaminase and gamma-glutamyl transferase were found to be high in both genders. Among females, the levels of alkaline phosphatase were significantly higher than the normal range in the study population (Table 2).

**Table 2:** Stratification of hematological parameters of the participants SD: standard deviation; BMI: body mass index; Hb: hemoglobin; TLC: total leucocyte count; ALT: alanine transaminase; GGT: gamma-glutamyl transferase; INR: international normalized ratio. * P-value determined using Student's t-test.

Parameters	Cumulative (mean ± SD)	Male (mean ± SD)	Female (mean ± SD)	p-value*	Range
Age (years)	38.41 ± 14.8	44.13 ± 14.4	34.19 ± 13.9	<0.001	-
BMI (kg/m^2^)	24.53 ± 4.20	26.3 ± 2.65	23.2 ± 4.66	<0.001	-
Serum amylase	114.56 ± 30.8	109.9 ± 30.5	118.1 ± 30.8	0.133	40-140 U/L
Hb (g/ dL)	11.99 ± 0.9	12.3 ± 0.78	11.76 ± 0.91	<0.001	-
TLC	5226.4 ± 3507.9	5136 ± 3538.8	5293.9 ± 3506.9	0.8	4000-11,000 cells per cmm
Platelets	189801.5 ± 135165.8	188870 ± 135915.4	190496.9 ± 135515.8	0.95	150,000-450,000 per microliter
ALT	44.2 ± 7.59	45.8 ± 7.18	43.01 ± 7.71	0.04	4-36 U/L
Bilirubin	16.5 ± 1.78	16.64 ± 1.92	16.45 ± 1.68	0.538	1.71-20.5 micromol/L
Alkaline phosphatase	146.5 ± 26.7	141.2 ± 24.3	150.5 ± 27.9	0.048	44-147 U/L
GGT	43.2 ± 10.05	42.5 ± 10.5	43.8 ± 9.75	0.477	8-38 U/L
INR	1.005 ± 0.05	1.009 ± 0.05	1.003 ± 0.05	0.494	-

**Figure 1:**
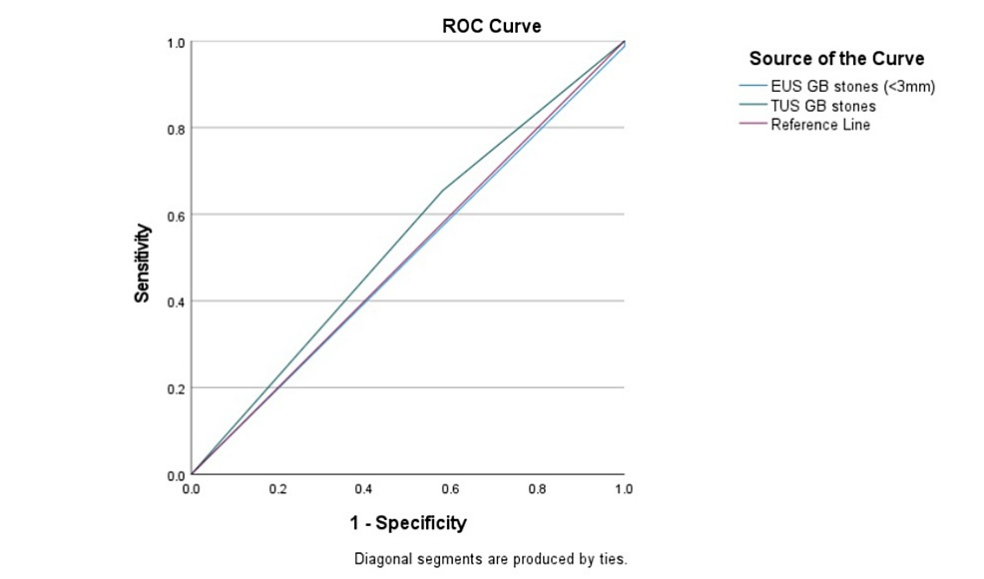
Assessment of modalities using the ROC curve (for gallstones) ROC: receiver operating characteristic; EUS: endoscopic ultrasound; TUS: transabdominal ultrasound; GB: gallbladder.

**Figure 2:**
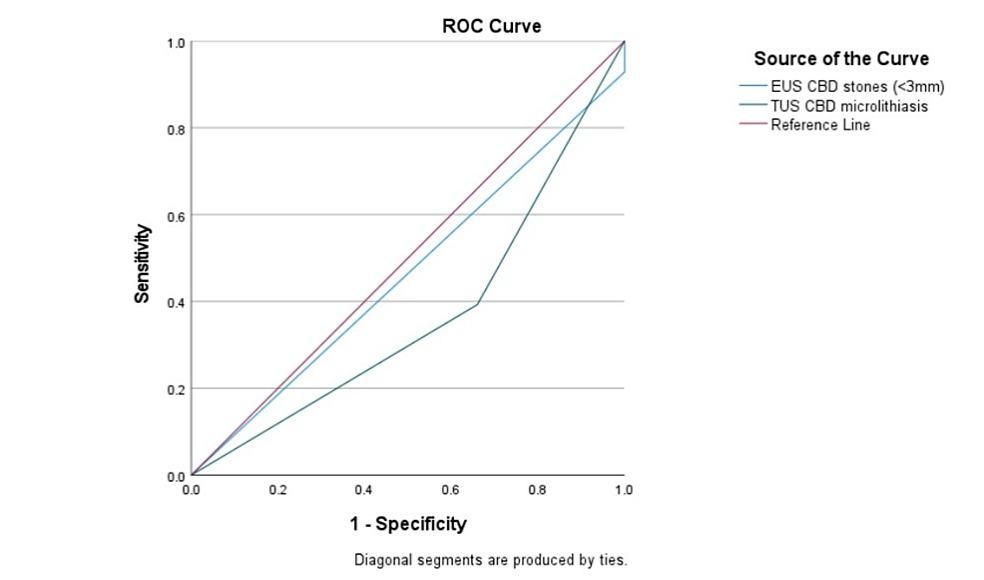
Assessment of modalities using ROC curve (for CBD stones) ROC: receiver operating characteristic; EUS: endoscopic ultrasound; TUS: transabdominal ultrasound; CBD: common bile duct.

Furthermore, the diagnostic precision of EUS in detecting gallstones and microlithiasis in the CBD was juxtaposed with TUS using ROC analysis, as shown in Table 3 and Figures 1, 2. The overall agreement between EUS and TUS was fair for CBD stones, with a kappa coefficient (95% confidence interval) of 0.214, and very weak for microlithiasis, with a kappa coefficient of -0.093.

**Table 3:** Efficacy of TUS and EUS in comparison with cholecystectomy and histology for gallbladder stones and ERCP for CBD stones were taken as the gold standard PPV: positive predictive value; NPV: negative predictive value; AUC: area under the curve; SD: standard deviation; CI: confidence interval; CBD: common bile duct; EUS: endoscopic ultrasound; TUS: transabdominal ultrasound.

Diagnostic modality		Sensitivity (%)	Specificity (%)	PPV (%)	NPV (%)	AUC	SD	p-value	Asymptotic 95% CI	
									Lower limit	Upper limit
Gallstones	EUS	98.8	100	100	98.8	0.494	0.05	0.906	0.392	0.596
	TUS	65.4	58	26.9	62.6	0.537	0.05	0.476	0.435	0.639
CBD stones	EUS	92.9	100	100	99.9	0.464	0.06	0.563	0.339	0.590
	TUS	39.3	66	53.6	47.9	0.366	0.06	0.03	0.249	0.484

## Discussion

Although conventional TUS serves as an initial investigation for patients with cholelithiasis and choledocholithiasis, it can also identify dilatation of the biliary tree in 85-95% of cases with confirmed obstruction [7]. EUS has demonstrated high sensitivity, up to 97%, in detecting CBD stones. This technique is regarded as safe and beneficial in identifying patients who would benefit from therapeutic ERCP, potentially avoiding unnecessary risks associated with ERCP when its need is uncertain [7].

Several studies have reported a mean age of 55-59 years with a genetic predisposition toward the male gender for this disease. However, in this study, the overall mean age of patients was 38.41 ± 14.8, and the females (57.3%) were found to be more affected, with a mean age of 34.19 ± 13.9 years. This finding aligns with the study conducted by Bansal and colleagues, where they concluded that the mean age of patients was 47.9 ± 18.87 years, with 54.7% female sufferers in the majority, compared to 45.3% male, contradicting the outcomes of multiple studies [9]. In contrast, Anwer et al. [10], Wang et al. [11], Netinatsunton et al. [12], and Khan et al. [13] found that 51.2%, 58.4%, 41.1%, and 58.5% of males were more affected and showed a high tendency to develop CBD stones than females, with mean ages of 50, 59, 55, and 50 years, respectively.

In this comparative study, EUS outperformed TUS in detecting CBD stones, demonstrating a specificity approaching 100%. TUS demonstrated a sensitivity of 65.4% and a specificity of 58% for detecting gallstones, and a sensitivity of 39.3% and a specificity of 66% for detecting CBD stones. These results indicate a high number of false negatives for TUS in this study. Similar findings were reported by Khan and colleagues, who also found a low sensitivity of 29.2% [13]. Additionally, Makmun et al. observed a low sensitivity range of 15-40% for detecting bile duct stones (choledocholithiasis) [14]. The literature attributes the low sensitivity of TUS to its subjective nature, effort in distinguishing air bubbles from other auditory shadows (as seen in cholangitis), intervention from abdominal fat and bowel gas shadows, and the tool's limitation in detecting smaller stones [15].

Furthermore, in our study, EUS exhibited a high diagnostic sensitivity of 92.9%, specificity of 100%, PPV of 100%, and NPV of 99.9% for bile duct stones, which showed the superiority of EUS over TUS. Netinatsunton and colleagues [12] conducted a cohort study that detected CBD stones in 59 patients using EUS, which were later confirmed by ERCP. In the high-risk category, EUS showed 96% sensitivity, 100% specificity, 100% PPV, and 77% NPV, respectively, in identifying typical bile duct stones [12]. In the intermediate-risk, EUS detected choledocholithiasis in 26 out of 73 subjects [12]. However, subsequent ERCP confirmed the presence of stones in only 24 of these 26 EUS-positive cases, resulting in two false positives. EUS exhibited a commendable overall sensitivity of 96% and specificity of 95.80% in discerning choledocholithiasis within this risk stratum. Its PPV was 92.30%, while its NPV stood at 97.90%. Post-procedural complications were notably absent in individuals undergoing EUS. Conversely, 6.3% of those undergoing ERCP encountered adverse events, such as mild pancreatitis, hemorrhage secondary to sphincterotomy, and sphincterotomy-associated perforations, necessitating prolonged hospitalization durations [13].

In a study by Chang and his coworkers [16], two patients were found to have CBD stones, while one had CBD sludge, all of which were only detected using EUS. Patients with microlithiasis in the CBD faced an elevated risk of developing complications such as biliary obstruction, cholangitis, and acute pancreatitis. Additionally, the high NPV of EUS was crucial in excluding choledocholithiasis [16]. Another study examined the characteristics of patients and the recurrence of EUS findings. The study concluded that higher levels of alanine transaminase or alkaline phosphatase upon admission, a prior history of cholecystectomy, and non-lithiasis findings on EUS were linked to a higher likelihood of experiencing a new episode of acute pancreatitis [17].

Limitations

The study has several limitations that need to be considered. Firstly, the selection of all study participants and EUS examinations were done by one experienced doctor, which introduces the possibility of selection bias in our study. This reliance on a single individual might affect the generalizability of the study findings. Secondly, it was a single-center study, and results may not be representative of a larger population. Lastly, post-procedure complications were not considered for this study.

## Conclusions

In conclusion, EUS is a valuable diagnostic tool; a less intrusive and extremely precise imaging technique for studying the pancreas and the biliary tree. Our study has demonstrated that EUS represents a significant advancement in diagnosing gallbladder and bile duct microlithiasis. It will also enhance the existing pool of data regarding the use and efficacy of EUS. Further research is necessary to ascertain the definitive role of EUS in gallstone management. This could help in identifying an effective diagnostic modality for individuals with an unclear cause of biliary colic.
